# Supplementary material for: One-Step Photochemical Immobilization of Aptamer on Graphene for Label-Free Detection of NT-proBNP
Source: Biosensors (Basel). 2022 Nov 23;12(12):1071. doi: 10.3390/bios12121071 (PMC9775241; doi:10.3390/bios12121071)
Supplement: Supplementary file 1 [file biosensors-12-01071-s001.zip › biosensors-1999910-supplementary.pdf]

biosensors

MDPI

Supplementary Information

One-Step Photochemical Immobilization of Aptamer on Graphene for Label-Free Detection of NT-proBNP

Nikita Nekrasov [1], Anastasiia Kudriavtseva [1], Alexey V. Orlov [2], Ivana Gadjanski [3], Petr I. Nikitin [2,*], Ivan Bobrinetskiy [1,3] and Nikola Ž. Knežević [3,*]

[1] Center for Probe Microscopy and Nanotechnology, National Research University of Electronic Technology, Moscow, 124498 Zelenograd, Russia
[2] Prokhorov General Physics Institute of the Russian Academy of Sciences, 119991 Moscow, Russia
[3] BioSense Institute—Research and Development Institute for Information Technologies in Biosystems, University of Novi Sad, 21000 Novi Sad, Serbia
* Correspondence: petr.nikitin@nsc.gpi.ru (P.I.N.); nknezevic@biosense.rs (N.Ž.K.)

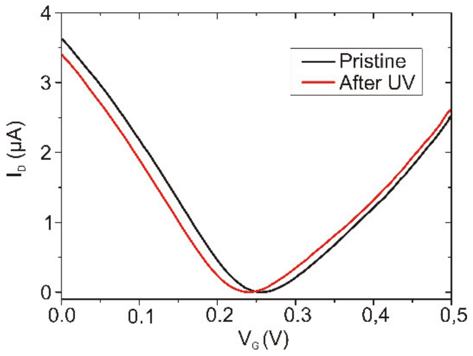

Copyright: © 2022 by the authors. Licensee MDPI, Basel, Switzerland. This article is an open access article distributed under the terms and conditions of the Creative Commons Attribution (CC BY) license (https://creativecommons.org/licenses/by/4.0/).

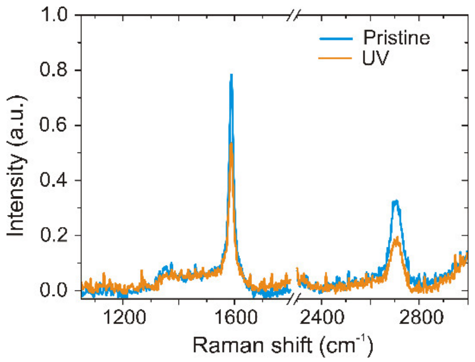

(**a**) (**b**)

Figure S1. Control GFET after UV irradiation in pure PBS. Change in CVCs for GFET after 10 min of UV irradiation in PBS solution (**a**). Dirac point shifts at 17 mV. Raman spectra of graphene before and after UV processing in PBS (**b**).

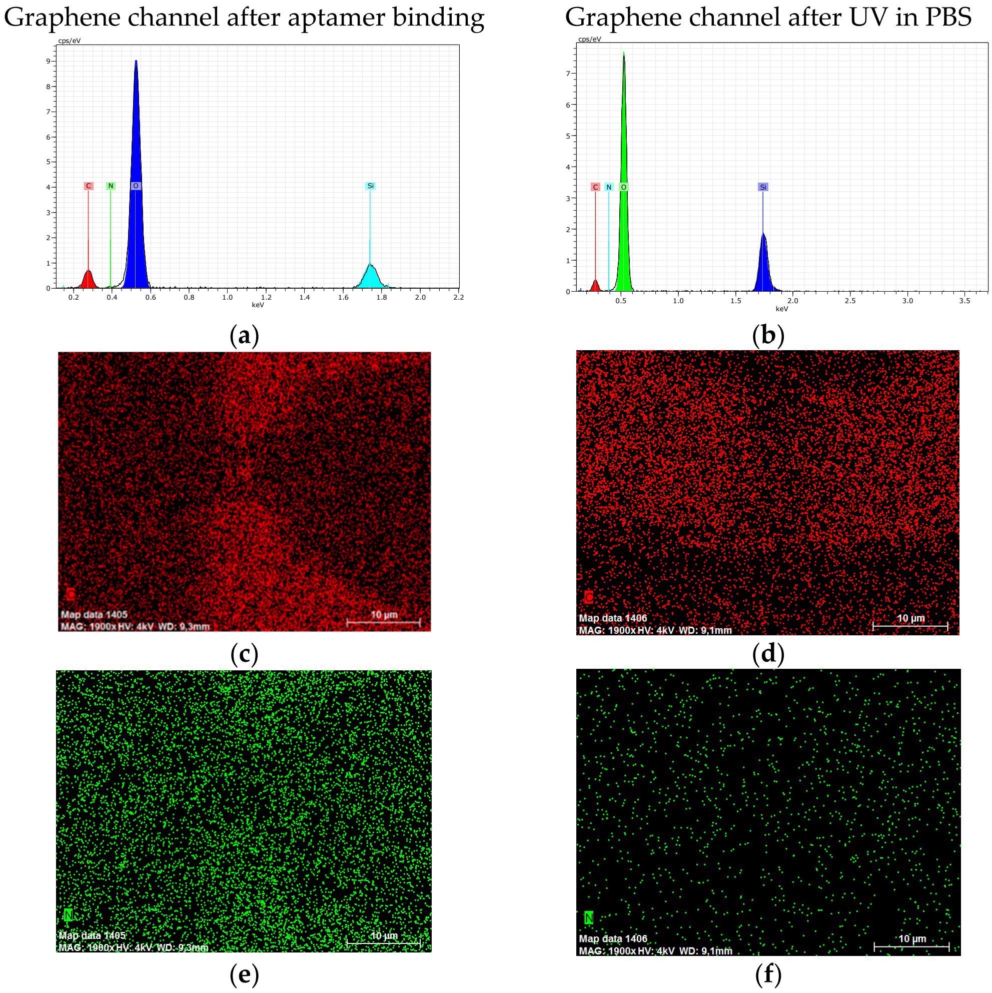

Figure S2. Energy dispersive spectroscopy characterization of modified graphene channels. Results of graphene surfaces after azide aptamer binding (left column) and after 5 min UV treatment without aptamer (right column). Red (**c**, **d**) and green (**e**, **f**) dots represent the parts covered with a carbon and nitrogen element, respectively. Scale bar: 10 µm.

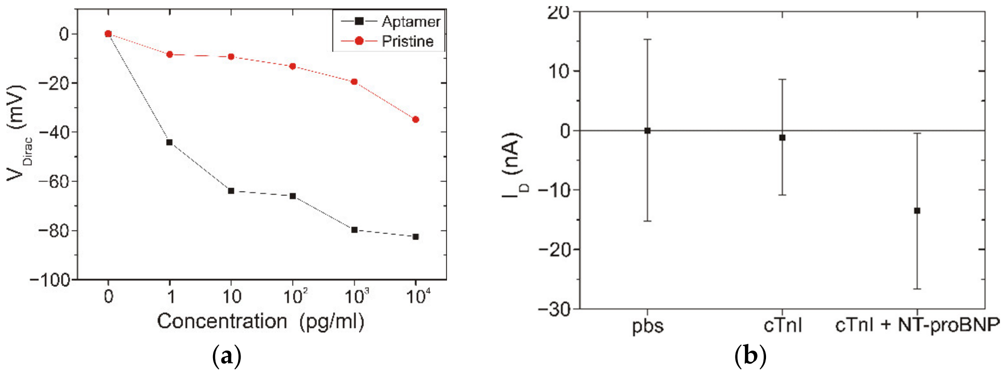

Figure S3. Control experiments on selectivity and specificity. (**a**) Dirac point shift for pristine (after 10 min UV treatment) and aptamer-modified graphene channels for increasing NT-proBNP concentrations. (**b**) ΔI_D for pure PBS, cTnI (3.5 ng/ml) and mix of 3.2 ng/ml cTnI + 1 ng/ml NT-proBNP for N20a aptamer-modified GET at a fixed gate voltage VG = 250 mV. The current values were treated to start at zero using the equation $\Delta I_D = I_D$-minimum(I_D).

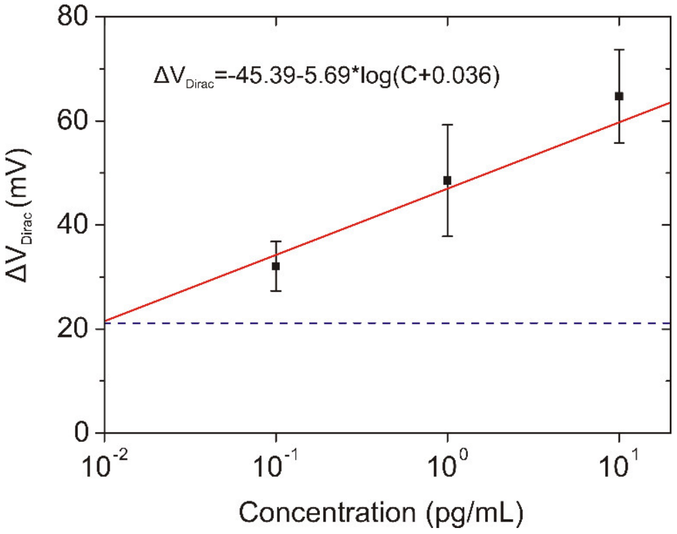

Figure S4. Calculation of LOD for GFET sensor, modified by azide-aptamer. Based on data at Figure 3b. The LOD was calculated as intersection of data from linear range and 3 times multiplied noise level of GFET.
